# Description of HIV-1 Group M Molecular Epidemiology and Drug Resistance Prevalence in Equatorial Guinea from Migrants in Spain

**DOI:** 10.1371/journal.pone.0064293

**Published:** 2013-05-22

**Authors:** Gonzalo Yebra, Miguel de Mulder, África Holguín

**Affiliations:** HIV-1 Molecular Epidemiology Laboratory, Dept. of Microbiology, Hospital Ramón y Cajal-IRYCIS and CIBERESP, Madrid, Spain; University of Amsterdam, The Netherlands

## Abstract

**Background:**

The HIV epidemic is increasing in Equatorial Guinea (GQ), West Central Africa, but few studies have reported its HIV molecular epidemiology. We aimed to describe the HIV-1 group M (HIV-1M) variants and drug-resistance mutations in GQ using sequences sampled in this country and in Spain, a frequent destination of Equatoguinean migrants.

**Methods:**

We collected 195 HIV-1M *pol* sequences from Equatoguinean subjects attending Spanish clinics during 1997-2011, and 83 additional sequences sampled in GQ in 1997 and 2008 from GenBank. All (n = 278) were re-classified using phylogeny and tested for drug-resistance mutations. To evaluate the origin of CRF02_AG in GQ, we analyzed 2,562 CRF02_AG sequences and applied Bayesian MCMC inference (BEAST program).

**Results:**

Most Equatoguinean patients recruited in Spain were women (61.1%) or heterosexuals (87.7%). In the 278 sequences, the variants found were CRF02_AG (47.8%), A (13.7%), B (7.2%), C (5.8%), G (5.4%) and others (20.1%). We found 6 CRF02_AG clusters emerged from 1983.9 to 2002.5 with origin in GQ (5.5 sequences/cluster). Transmitted drug-resistance (TDR) rate among naïve patients attended in Spain (n = 144) was 4.7%: 3.4% for PI (all with M46IL), 1.8% for NRTI (all with M184V) and 0.9% for NNRTI (Y188L). Among pre-treated patients, 9/31 (29%) presented any resistance, mainly affecting NNRTI (27.8%).

**Conclusions:**

We report a low (<5%) TDR rate among naïve, with PI as the most affected class. Pre-treated patients also showed a low drug-resistance prevalence (29%) maybe related to the insufficient treatment coverage in GQ. CRF02_AG was the prevalent HIV-1M variant and entered GQ through independent introductions at least since the early 1980s.

## Introduction

The human immunodeficiency virus type 1 group M (HIV-1M), responsible for the HIV pandemic, originated from a zoonotic transmission from chimpanzees into humans in Cameroon [1]. For decades, the virus evolved and diversified into different lineages in the Congo River basin, where the highest viral diversity circulates [2]. Today, HIV-1M is sub-divided into 9 pure subtypes (A-D, F-H, J and K) and recombinants between them (http://www.hiv.lanl.gov/content/sequence/HIV/CRFs/CRFs.html): 58 circulating recombinant forms (CRFs) and multiple unique recombinant forms (URFs).

Equatorial Guinea (GQ), a Spanish colony until 1968, is a small country located in West Central Africa between Cameroon and Gabon. The estimated number of people living with HIV in GQ (20,000 in 2009) increases annually, and antiretroviral treatment (ART) only reached 48% of them in 2011 [3]. Despite its location, close to the epicenter of HIV-1 pandemic, only two local studies [4], [5] have so far reported the HIV molecular epidemiology in GQ. Ortiz et al. included 76 HIV-1M *env* gene (gp41) sequences obtained in 1999 from general HIV-infected population. Djoko et al. evaluated 41 HIV-1M *pol* sequences sampled in 2008 from military personnel in Malabo −the capital city. Both studies revealed CRF02_AG as the prevalent HIV-1M variant, as happens in the neighboring countries [6].

The latter also reported a rate of transmitted antiretroviral drug resistance (TDR) of 4.2%. This rate represents the percentage of naïve patients infected with HIV variants carrying drug resistance mutations, and threatens the effectiveness of ART. Recent studies report an increasing TDR prevalence in low- and middle-income countries following ART scale-up [7]. This rise especially affects sub-Saharan Africa, driven by the resistance to non-nucleoside reverse transcriptase inhibitors (NNRTI) [8], [9]. The TDR rate in Cameroon is around 8–9% [10], [11] and lower than 5% in Gabon [12]. HIV molecular epidemiology studies are crucial in these areas to prevent the widespread transmission of drug resistance, especially those lacking systematic surveillance as GQ.

We aimed to describe the circulating HIV-1M variants and the presence of antiretroviral drug resistance mutations in Equatorial Guinea. To achieve this, we applied phylogenetic methods to a combination of viral sequences sampled in Spain (frequent destination of Equatoguinean migration) and publicly available sequences sampled in the sub-Saharan country.

## Materials and Methods

### Study Population

All the HIV-infected subjects coming from Equatorial Guinea with available HIV-1M *pol* sequences, and attended in Spanish HIV/AIDS clinics (mainly in Madrid) were collected. They were 195 adult and pediatric patients sampled between 1997 and 2011. Most sequences were previously reported [13]–[16], while 37 were unpublished. In most cases (136, 69.7%) they included the complete protease (PR, 297nt) and partial reverse transcriptase (RT, 860nt of mean length). In 58 (29.7%) and 1 (0.5%) cases, only the PR or the RT sequence was available, respectively. In addition, all the HIV-1 *pol* sequences (n = 83) sampled in Equatorial Guinea and available in the GenBank (www.ncbi.nlm.nih.gov/genbank) were retrieved. They belonged to two sequences batches sampled in 1997 (not published) and 2008 [5]. GenBank accession numbers of the sequences included are provided in **Accession S1**.

### HIV-1M Subtyping

HIV-1M variants were re-classified by phylogenetic analysis of the 278 *pol* sequences, including recombinants not available at publishing time. Representative sequences of the HIV-1M 9 subtypes and 49 CRFs downloaded from Los Alamos HIV sequence database (LANL-DB, http://www.hiv.lanl.gov) were used as references. Sequences were aligned manually, and initial neighbor-joining trees were built using MEGA5 (http://www.megasoftware.net) under the Kimura two-parameter model with 1,000 bootstrap re-sampling. Subtypes were further confirmed in approximately maximum-likelihood (ML) phylogenetic trees constructed under the general time reversible model of nucleotide substitutions with gamma-distributed heterogeneity rate (GTR+Γ) using FastTree v2.1.3 (http://www.microbesonline.org/fasttree). The topology robustness was tested by likelihood-based local branch support. In sequences not ascribed to any known subtype or CRF, recombination analyses were performed using SimPlot v3.5.1 and Recombination Detection Program (RDP, v3alpha44).

In the CRF02_AG subset, we searched monophyletic clusters (associations of ≥3 samples) that involved only sequences from GQ in ML trees that included all CRF02_AG sequences from LANL-DB (n = 2,652) constructed as described above with FastTree v2.1.3.

The time to the most recent common ancestor (TMRCA) of these Equatoguinean CRF02_AG clusters was estimated using Bayesian Markov chain Monte Carlo (MCMC) inference with the program BEAST v1.6.1 (http://beast.bio.ed.ac.uk). A lognormal distributed prior was placed on the estimated number of nucleotide substitution per site per year (mean = 2.5 × 10^−3^; standard deviation = 0.5). The MCMC chain ran for 10^8^ generations, sampling estimates every 10,000th generation. The uncorrelated lognormal molecular clock model and the SRD06 nucleotide substitution were selected. These parameters were chosen based on previous estimates for CRF02_AG datasets [17]. The tree samples were used to generate a maximum clade credibility (MCC) tree, after a 10% burn-in, using TreeAnnotator v1.6.1. Those Equatoguinean CRF02_AG clusters with a high clade support in both ML and Bayesian trees (≥95%) were chosen as definitive.

### Drug Resistance

For HIV sequences from antiretroviral-naïve patients, the TDR prevalence was defined following the World Health Organization mutation list [18] using the Calibrated Population Resistance tool (http://cpr.stanford.edu). For antiretroviral-exposed patients, substitutions considered in the 2011 International AIDS Society-USA (IAS-USA) mutation list [19] were manually identified.

### Ethics Statement

This study was part of a project approved by the review board of the Hospital Ramón y Cajal Clinical Research Ethical Committee. It was designed to protect the rights of all subjects involved under the appropriate local regulations. To maintain subject confidentiality, a unique ID number was assigned to each specimen, and written consent obtained for each patient by clinicians. For the minors involved in the study, written consent was provided by their parents or legal representatives.

## Results

### Equatoguinean HIV-infected Patients in Spain

The main characteristics of these patients are shown in **Table 1**. Most subjects (61.1%) of this cohort were women, and were infected with HIV due to heterosexual risk practices, in concordance with the general situation in sub-Saharan Africa [20]. Sorting patients according to the sample periods shown in Table 1, we observed a significant increase in the proportion of men: 13.6% in 1997–2001, 44.6% in 2002–2006 and 53.5% in 2007–2011 (*P* = 0.0002). We found no apparent trends in the remaining analyzed features across time. The epidemiological information was not available in the 83 sequences retrieved from GenBank and sampled in Equatorial Guinea.

**Table 1:** Epidemiologic characteristics at sampling time of the 195 Equatoguinean HIV-infected patients followed in Spain (1997–2011).

Characteristic	n	% (95% CI)
Gender		
Male	70	38.9 (32.1–46.2)
Female	110	61.1 (53.8–67.9)
Unknown	15	–
Population		
Adult	186	95.4 (91.5–97.5)
Pediatric	9	4.6 (2.4–8.5)
Risk practice		
Heterosexual	128	87.7 (81.3–92.1)
MSM	1	0.7 (0.1–3.8)
IDU	2	1.4 (0.4–4.9)
Vertical	9	6.2 (3.3–11.3)
Transfusion/others	6	4.1 (1.9–8.7)
Unknown	49	–
ART experience		
Naïve	148	82.7 (76.5–87.5)
Pre-treated	31	17.3 (12.5–23.5)
Unknown	16	–
Sampling period		
1997–2001	44	22.6 (17.3–28.9)
2002–2006	105	53.8 (46.8–60.7)
2007–2011	46	23.6 (18.2–30.0)
Mean sampling year (± SD)	2003.96±3.26	–
Mean HIV viral load (log ± SD) a	4.3±1.0	–

CI, confidence interval; MSM, men who have sex with men; IDU, injecting drug users; ART, antiretroviral treatment; SD, standard deviation. For naïve and pre-treated patients, the mutations lists from the World Health Organization [18] and from the International AIDS Society-USA [19] were considered, respectively. This information was not available for the 83 HIV-1M sequences retrieved from GenBank and sampled in Equatorial Guinea.

a Viral load was only available in 126 cases.

### HIV-1M Variants Circulating in Equatorial Guinea

**Table 2** shows the distribution of HIV-1M variants among the 278 *pol* sequences from Equatoguinean patients, where the recombinant CRF02_AG (47.8%) was predominant. Pure subtypes infected 122 (43.9%) patients, and other CRFs were found in 19 (6.8%) cases. Four sequences were classified as URF (URF_JU, URF_BH, URF_02D and URF_BG) using RDP and SimPlot. Of note, the frequency of subtype B was significantly higher in Equatoguinean subjects sampled in Spain than in those sampled in EQ (9.7% vs. 1.2%, *P* = 0.012).

**Table 2:** HIV-1 group M variants infecting the 278 patients from Equatorial Guinea.

HIV-1M variant	Total (%)	Sampled inSpain (%)	Sampled inGQ (%)
Pure subtypes	122 (43.9)	84 (43.1)	38 (45.8%)
A	38 (13.7)	24 (12.3)	14 (16.9)
B a	20 (7.2)	19 (9.7)	1 (1.2)
C	16 (5.7)	10 (5.1)	6 (7.2)
D	14 (5.0)	9 (4.6)	5 (6.0)
F	11 (4.0)	7 (3.6)	4 (4.8)
G	15 (5.4)	9 (4.6)	6 (7.2)
H	8 (2.9)	6 (3.1)	2 (2.4)
Recombinants	156 (56.1)	111 (56.9)	45 (54.2)
CRF02_AG	133 (47.8)	92 (47.2)	41 (49.4)
CRF06_cpx	4 (1.4)	3 (1.5)	1 (1.2)
CRF09_cpx	1 (0.4)	0 (0.0)	1 (1.2)
CRF11_cpx	7 (2.5)	6 (3.1)	1 (1.2)
CRF13_cpx	3 (1.1)	3 (1.5)	0 (0.0)
CRF18_cpx	1 (0.4)	1 (0.5)	0 (0.0)
CRF22_01A1	3 (1.1)	2 (1.0)	1 (1.2)
URF b	4 (1.4)	4 (2.1)	0 (0.0)
All	278	195	83

GQ, Equatorial Guinea; CRF, circulating recombinant form; URF, unique recombinant form. Sequences obtained in Spain were sampled between 1997 and 2011, and those obtained in Equatorial Guinea in 1997 (n = 35) (not published) or 2008 (n = 48; [5]).

a Subtype B prevalence was significantly higher among patients sampled in GQ than in Spain (*P* = 0.012, chi-square test).

b The four URF were URF_JU, URF_BH, URF_02D and URF_BG.

The new analysis of the sequences permitted to update their classification, especially among recombinants. We re-classified as CRF02_AG 21 sequences initially assigned to subtype G when they were sampled [21], [22], 1 CRF11_cpx reported as subtype J [22], 1 CRF13_cpx and 1 CRF18_cpx also reported as subtype G [21] and 1 CRF22_01A1 described as subtype A [16]. The recombination analyses revealed that 2 sequences isolated in 1999 and 2000 and reported as pure subtypes G and J [21] were URF_BG and URF_JU, respectively.

### CRF02_AG Cluster Analysis

**Figure 1:**
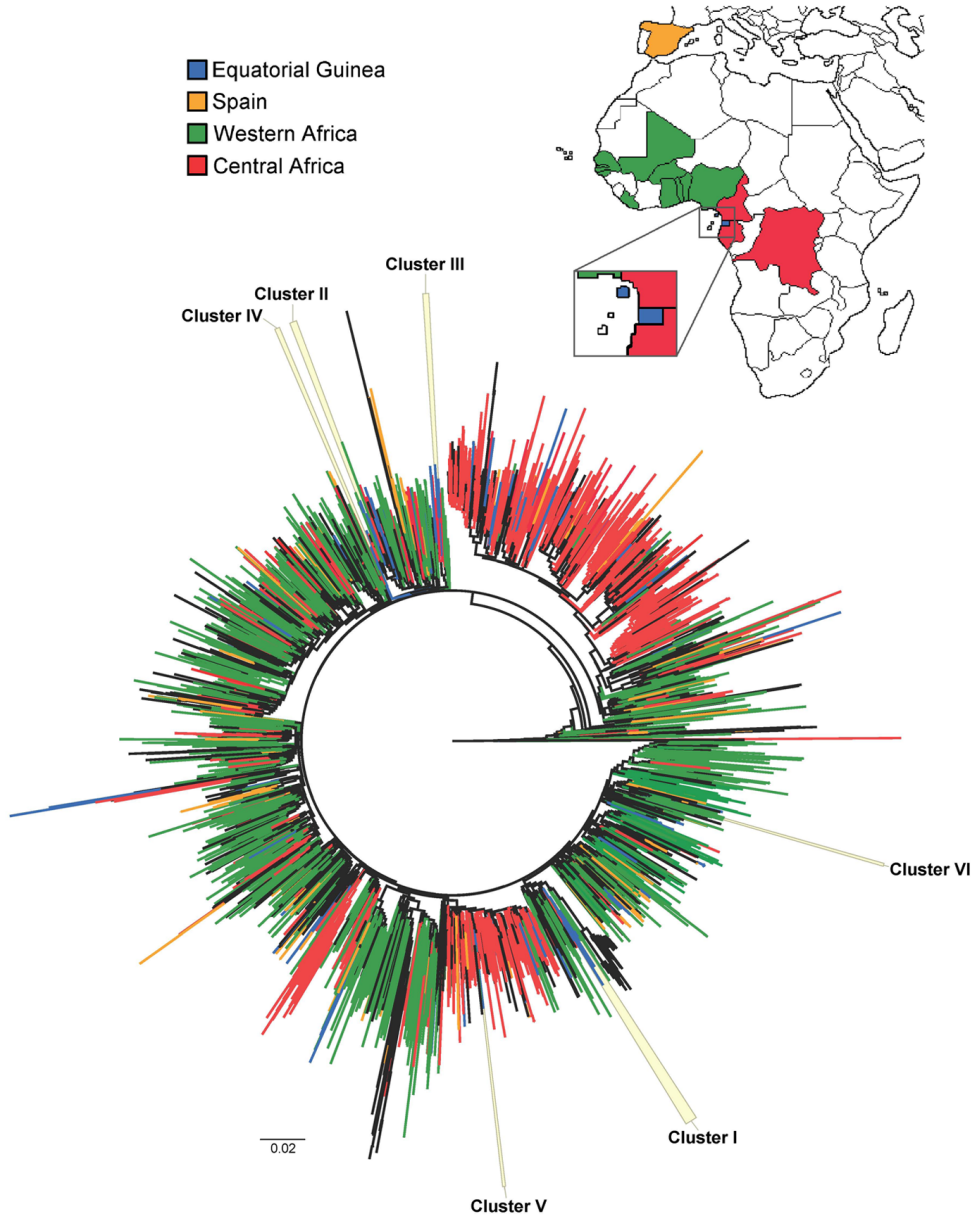
Maximum likelihood tree of the 133 CRF02_AG HIV-1 *pol* sequences with 2652 CRF02_AG sequences retrieved from Los Alamos Database. Tree constructed under the GTR+Γ model of nucleotide substitution using FastTree v2.1.3 (see Methods) from an 1130nt alignment. Branch colors indicate the origin of the sequences (with the same color code in the map). In blue, sequences from Equatoguinean patients (n = 133). In orange, CRF02_AG sequences from Spain (n = 158). In green, CRF02_AG sequences from Western Africa: Nigeria (n = 391), Mali (n = 235), Senegal (n = 185), Ghana (n = 180), Burkina Faso (n = 97), Benin (n = 77), Togo (n = 58), Liberia (n = 4), the Gambia (n = 2) and Guinea-Bissau (n = 2). In red, CRF02_AG sequences from Central Africa: Cameroon (n = 711), Gabon (n = 72), and Democratic Republic of the Congo (n = 7). In black, CRF02_AG sequences from other regions. The 6 monophyletic clusters from Equatorial Guinea further assessed using BEAST are highlighted in yellow and labeled in accordance to **Table 3**.

In the general ML tree that included the 133 CRF02_AG sequences of the study population and 2,652 worldwide CRF02_AG sequences (**Figure 1**), we found 7 monophyletic clusters including exclusively Equatoguinean CRF02_AG sequences with >95% of statistical support. The geographic origin of the 2,652 CRF02_AG sequences from LANL-DB is described in the legend of **Figure 1**. The further analysis with BEAST included a 92 sequence alignment: the CRF02_AG involved in the putative Equatoguinean clusters (n = 39), their closest relatives among the CRF02_AG from LANL-DB according to the ML tree (n = 15), and reference CRF02_AG sequences with diverse sampling dates (n = 38) in order to include at least one sequence per year from 1990 when available. The sampling times of the sequence dataset included in the BEAST analysis are shown in the supplementary **Table S1**.

The Bayesian MCC tree (**Figure S1**) showed that 6 of the 7 putative clusters were supported by a posterior probability of ≥0.95 (**Table 3**). Therefore, these 6 clusters were considered as definitive due to their high statistical support. They included 33 Equatoguinean sequences (mean = 5.5 sequences/cluster). All but one (cluster VI) clusters included sequences sampled in both Spain and GQ. The dates of their most recent common ancestors (MRCA) ranged from 1983.9 to 2002.5. Five of the 6 clusters were included among sequences sampled in Western Africa, and one (cluster V) was related to sequences sampled in Central Africa (**Figure 1**).

**Table 3:** Characteristics of the monophyletic clusters of HIV-1M CRF02_AG *pol* sequences observed in the Equatoguinean study population.

Cluster	Sequences (ES; GQ)	Sampling year range	MRCA date (95% HPD)	Support (LBS; PP)
I	11 (8; 3)	1997–2006	1983.89 (1979.90–1989.76)	0.95; 0.99
II	6 (5; 1)	1997–2010	1991.73 (1987.38–1995.26)	0.99; 1
III	6 (3; 3)	1997–2008	1992.31 (1988.02–1996.19)	0.99; 1
IV	4 (3; 1)	2007–2009	1993.25 (1983.95–2000.89)	0.96; 1
V	3 (2; 1)	2001–2008	1994.52 (1990.26–1998.71)	0.98; 1
VI	3 (3; 0)	2004–2005	2002.52 (2000.32–2004.21)	0.98; 1

ES, sequences sampled in Spain; GQ, sequences sampled in Equatorial Guinea; MRCA, most recent common ancestor; HPD, highest posterior density interval; LBS, local branch support in the maximum likelihood tree; PP, posterior probability in the Bayesian dated tree.

### HIV-1M Drug Resistance

Among the 195 sequences collected in Spain from Equatoguinean subjects, 148 (75.9%) corresponded to antiretroviral-naïve patients and 31 (15.9%) to treatment-experienced subjects, with unknown treatment experience in 16 (8.2%) patients. We found at least one transmitted drug-resistance mutation (TDR) in 7 of the 148 (4.7%) naïve patients following the World Health Organization mutations list [18]. TDR was 6.7% (6/90) in women vs. 1.8% (1/56) in men (*P* = 0.2). **Table 4** shows the TDR rate according to the antiretroviral class affected. In 5 (3.4%) subjects, the mutations affected PR inhibitors (PI), with M46I/L substitution in all cases. Another 2 (1.8%) presented the M184V mutation to nucleoside reverse transcriptase inhibitors (NRTI). One of the latter also presented a drug-resistance mutation to non-nucleoside reverse transcriptase inhibitors (NNRTI). No triple-class resistance was found. Among the 31 ART-experienced patients, 9 (29%) presented any drug-resistance mutation according to the IAS-USA list [19]. The highest rate (27.8%) was found for NNRTI-resistance due to mutation K103N in 3 of the 7 cases. The PI-resistance mutations found were M46L (4 cases), and L90M and I84V (3 cases each). For NRTI-resistance, the mutation M184V was found in all 3 cases. We observed a fluctuation in TDR rates in the three periods shown at Table 1∶9.7% (3/31) in 1997–2001, 2.5% (2/81) in 2002–2006 and 5.5% (2/36) in 2007–2011, probably due to the small sample size in each one.

**Table 4:** Prevalence of drug resistance mutations in 148 antiretroviral-naïve and 31 pre-treated HIV-1-infected patients from Equatorial Guinea and followed in Spain.

	Naïve patients (148)			Pre-treated patients (31)		
ART class	TDR seqs.	No. seqs.	% (95% CI)	DRM seqs.	No. seqs.	% (95% CI)
**Any**	7	148	4.7 (2.3–9.4)	9	31	29.0 (16.1–46.6)
**PI**	5	148	3.4 (1.4–7.7)	5	31	16.1 (7.1–32.6)
**NRTI**	2	108	1.8 (0.5–6.5)	3	18	16.7 (5.8–39.2)
**NNRTI**	1	108	0.9 (0.2–5.1)	5	18	27.8 (12.5–50.9)

ART, antiretroviral; No, number; seqs., sequences; DRM, drug resistance mutations; CI, confidence interval; PI, protease inhibitors; NRTI, nucleoside reverse transcriptase inhibitors; NNRTI, non-nucleoside reverse transcriptase inhibitors. For naïve and pre-treated patients, the mutations lists from the World Health Organization [18] and from the International AIDS Society-USA [19] were considered, respectively.

**Table 5** shows the main epidemiological and virological features of the 7 drug-naïve and the 9 drug-experienced patients carrying viruses harboring resistance mutations. Of note, most naïve (6/7; 85.7%) and pre-treated (6/9 cases; 66.7%) patients carrying drug-resistant viruses were adult women. They were mainly infected by CRF02_AG (2/7 and 4/9, respectively) and subtype B (2/7 and 3/9 cases, respectively).

**Table 5:** Epidemiological and virological characteristics of the 7 antiretroviral-naïve and the 9 antiretroviral-experienced patients infected by HIV-1M variants harboring drug resistance mutations.

#	ART	Population	HIV-1Mvariant	Gender	Riskpractice	Samplingyear	HIV VL(log)	TDR-PI	TDR-NRTI	TDR-NNRTI
1	Naïve	Adult	B	F	Htsex	1999	2.5	M46L, L90M	NA	NA
2	Naïve	Adult	B	F	NA	1999	4.3	M46L, I84V	NA	NA
3	Naïve	Adult	C	F	Htsex	1999	1.8	M46I	NA	NA
4	Naïve	Adult	CRF02_AG	F	NA	2005	3.6	–	M184V	–
5	Naïve	Adult	CRF11_cpx	F	Htsex	2006	2.9	–	M184V	Y188L
6	Naïve	Adult	A1	F	Htsex	2007	2.7	M46L	–	–
7	Naïve	Pediatric	CRF02_AG	M	Vertical	2007	NA	M46L	–	–
8	Treated	Adult	B	F	Htsex	1999	2.3	M46L	NA	NA
9	Treated	Adult	B	F	Htsex	1999	2.3	M46L, L90M	NA	NA
10	Treated	Adult	B	F	Htsex	1999	2.3	M46L, I84V, L90M	NA	NA
11	Treated	Adult	CRF02_AG	F	Htsex	2003	4.0	F53L, I84V, L90M	M184V	K103N
12	Treated	Adult	F2	M	Htsex	2006	NA	–	–	V106I
13	Treated	Adult	D	M	Htsex	2008	5.0	–	–	V90I
14	Treated	Pediatric	CRF02_AG	F	Vertical	2009	NA	–	M184V	–
15	Treated	Adult	CRF02_AG	M	Htsex	2009	6.0	–	–	K103N
16	Treated	Pediatric	CRF02_AG	F	Vertical	2010	NA	M46L, I84V	L74V, Y115F, M184V	K103N, G190A

#, number of patient; ART, antiretroviral treatment; VL, viral load; TDR, transmitted drug resistance mutations; PI, protease inhibitors; NRTI, nucleoside reverse transcriptase inhibitors; NNRTI, non-nucleoside reverse transcriptase inhibitors; F, female; M, male; Htsex, heterosexual; NA, data not available; Dash, no mutation found.

Regarding the sequences retrieved from GenBank and obtained in Equatorial Guinea, the antiretroviral status was only available for the 41 samples described in Djoko et al., which were obtained from naïve HIV-infected population in Malabo. Among them, TDR was 4.9% according to the WHO mutations list.

## Discussion

Despite being located in the River Congo basin, where the HIV epidemics originated [1], only two works [4], [5] have provided data about the circulation of different HIV strains and drug resistance in Equatorial Guinea. Here, we expand and complete the information available about the HIV Equatoguinean epidemics combining the use of HIV-1M *pol* sequences from immigrants in Spain (the main host developed country for Equatoguinean migration) and from patients sampled in Equatorial Guinea. This is the larger study covering transmitted drug resistance in this country and the first one analyzing general HIV-infected population and dating the introduction of CRF02_AG.

### CRF02_AG Recombinant is Prevalent in Equatorial Guinea

We confirm that recombinant CRF02_AG, the prevalent HIV-1M variant in Western Africa [6], accounts for a half of infections in Equatorial Guinea (**Table 2**). This is also the most frequent non-B variant in Spain among adult [23] and pediatric [15] HIV-infected population. The sequences’ re-analyses identified CRF02_AG isolates classified as subtype G when they were described using only protease sequences [21]. We also found CRF11_cpx, CRF13_cpx, CRF18_cpx and CRF22_01A1 sequences initially described as pure subtypes [16], [22]. These complex variants are found in Spain among immigrants and rarely in autochthonous population [14], [23]. Recombinants might be underestimated in studies performed in periods that lacked reference sequences published afterwards. Thus, periodic re-assessments of HIV classification with updated CRFs might improve the subtyping. In addition, modern computer tools permit to identify previously unnoticed unique recombinant forms when phylogenetic analyses are inconclusive.

### CRF02_AG Introduction in Equatorial Guinea

The Bayesian MCMC analysis using BEAST revealed 6 monophyletic, highly statistically supported clusters of Equatoguinean CRF02_AG sequences. This could indicate that this variant entered Equatorial Guinea through several independent introductions occurred at least since the early 1980s, and not through a single introduction event. A previous study proposed that CRF02_AG was introduced in Equatorial Guinea from Cameroon [24], but our results showed that only one of the 6 fell within Central African clades, being the rest related to Western African clades. In many cases, sequences from both regions were interspersed in the analysis (**Figure 1**), which suggests a frequent circulation of CRF02_AG between countries, as others have also indicated [25]. In both Western and Central Africa the presence of this recombinant, originated at least in the early 1970s [24], has increased in the last years [6].

On the other hand, the observed clustering could also be explained by specific transmission networks, although the available epidemiological information collected in the patients’ clinical records does not point to the presence of any of these networks. In addition, as previously commented, all clusters but one (cluster VI) included both CRF02_AG sequences sampled in Spain and in Equatorial Guinea, which minimizes the chance of a local transmission network. Despite these explanations, this alternative possibility cannot be completely ruled out.

### Low Rate of Resistance among HIV-1M-infected Equatoguineans

We observed a TDR rate of 4.7% among the 148 antiretroviral-naïve Equatoguineans attending Spanish HIV/AIDS clinics, a figure close to the limit between low and moderate drug-resistance (5%) according to the World Health Organization [26]. This rate is very similar to that found in the only TDR study performed in Equatorial Guinea (4.9%), which screened military personnel in Malabo [5]. Both are lower than in other sub-Saharan countries, including the neighboring Cameroon, with a TDR around 9% [10], [11]. Since ART still reaches only 48% of the HIV-infected population in need of therapy in GQ [3], a lower emergence of resistance than in countries with an older ART scale-up is expected [7].

The highest TDR rate was found for PI (3.4%), led by the presence of the substitution M46I/L in all cases. This was also the most frequent PI-resistance mutation among pre-treated patients, and it was previously reported among antiretroviral-naïve military personnel living in Equatorial Guinea [5]. This high prevalence contrasts with the rare administration of PI in Equatorial Guinea, where the first line treatment includes the NNRTI efavirenz [3]. For transmitted NRTI-resistance, the low presence found (with the predominance of change M184V) agrees with the study of Djoko et al., although they only found the substitution D67N as transmitted NRTI-resistance mutation [5].

Studies in Uganda [7], [27] have related the expanded use of NNRTI as peripartum prophylaxis among HIV-infected pregnant women to a higher population-wide prevalence of NNRTI-resistance mutations. Thus, the low presence of transmitted NNRTI-resistance mutations in Equatoguineans compared with other sub-Saharan populations [8] could result from the infrequent administration of this prophylaxis in Equatorial Guinea (only in 19% of HIV-infected pregnant women in 2010) [3].

In treated Equatoguinean patients attended in Spain, we found a presence of drug-resistance mutations rate of 29%, with NNRTI as the most affected drug class (27.8%). This lower prevalence than in other sub-Saharan antiretroviral-exposed cohorts [28] could reflect a poor adherence as observed among sub-Saharans living in Spain [29], who present great rates of follow-up losses. This has also been reported in Africa [30], caused by the low accessibility of antiretroviral drugs, as happens in Equatorial Guinea, with an antiretroviral coverage of 48% in 2011. In Equatorial Guinea, the use of antiretroviral drugs started in a regular basis in 2005. Thus, previous sporadic treatments and interrupted exposition to drugs could have caused the appearance of resistance mutations that jeopardizes the success of a future systematic antiretroviral programme. Unfortunately, in most cases the information on specific drug exposure for the pre-treated patients was unavailable, which prevent us from a further interpretation of these data.

### HIV Infection Place

The phylogenetic analyses showed that HIV-1M sequences sampled in Spain and/or Equatorial Guinea were interspersed, which would suggest that most of the 195 Equatoguinean patients with clinical follow up in Spain were infected in their country of origin. However, we cannot rule out that some of them could have been infected in Spain. This hypothesis would be supported by the overrepresentation of subtype B (the predominant variant in Spain [23] but infrequent in Central Africa) among samples taken in this host country versus those taken in Equatorial Guinea; and by the higher prevalence of transmitted PI-resistance in these subtype B-infected Equatoguineans sampled in Spain (where treatment is universally available) than in other variants of the study cohort.

Conversely, an evidence for an infection happened in Africa would be the different mutation pattern found in Equatoguineans sampled in Spain than in a study of general, naïve patients [14] (autochthonous and African and non-African foreigners) living in Spain between 1996 and 2010. Thus, the PI-resistance mutation M46L, present in all subtype B viruses with TDR (**Table 5**), was rare (<1%) among the subtype B-infected population living in Spain, where L90M was predominant. In addition, if the Equatoguinean patients had been infected in Spain, higher rates of NRTI- and, especially, NNRTI-resistance would have been expected.

In conclusion, the rising HIV drug-resistance transmission in sub-Saharan Africa following antiretroviral rollout highlights the need of periodical surveillance studies to monitor and prevent the resistance emergence, essential information to design or continue the implementation of ART programmes. These studies should be performed among infected people living in the study region. However, in countries lacking surveillance reports (such as Equatorial Guinea), our approach describing the HIV molecular epidemiology of low-income regions from a developed host country can provide any knowledge about the HIV epidemic in specific areas.

## Supporting Information

Figure S1 **Maximum clade credibility tree of the 92 CRF02_AG** ***pol*** **sequences included in the BEAST analysis.** The definitive clusters are highlighted in yellow and labeled in accordance to **Table 3** **.** The asterisk indicates nodes supported by a posterior probability of ≥0.95. The horizontal axis is expressed in calendar years. Branch colors indicate the origin of the sequences: blue, sequences from Equatoguinean patients (n = 39); green, sequences from Western Africa (n = 21); red, sequences from Central Africa (n = 17); black, other regions (n = 15).
(TIF)

Table S1 **Sampling dates of the 92 CRF02_AG** ***pol*** **sequences included in the BEAST analysis. **n, number of sequences.
(DOCX)

Accession S1 **GenBank accesion numbers of the 278 HIV-1** ***pol*** **sequences included in this study.**
(DOCX)
