# Supplementary figures and images for: Description of HIV-1 Group M Molecular Epidemiology and Drug Resistance Prevalence in Equatorial Guinea from Migrants in Spain
Source: PLoS One. 2013 May 22;8(5):e64293. doi: 10.1371/journal.pone.0064293 (PMC3661467; doi:10.1371/journal.pone.0064293)

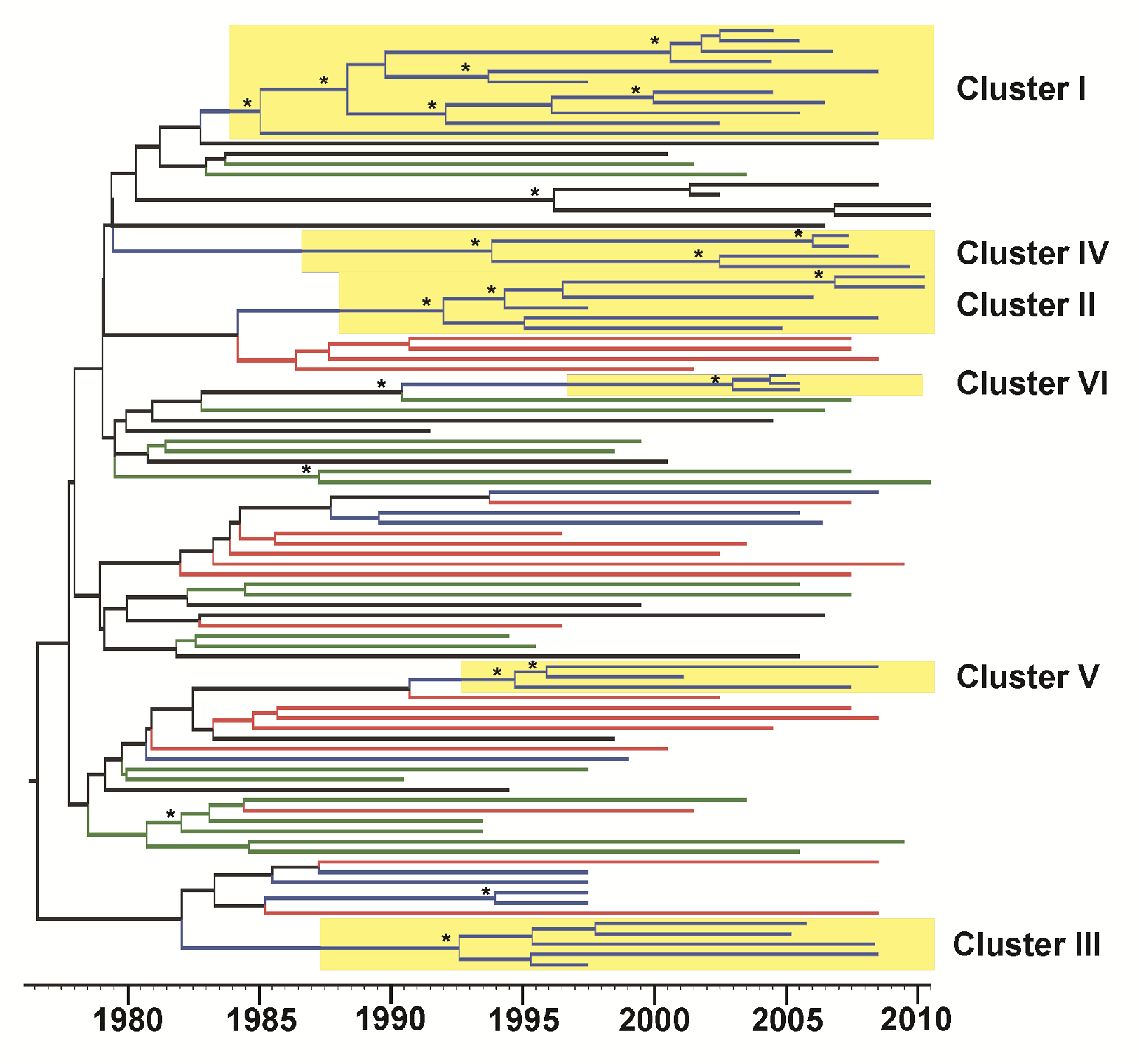

Supplement: Figure S1 — Maximum clade credibility tree of the 92 CRF02_AG pol sequences included in the BEAST analysis. The definitive clusters are highlighted in yellow and labeled in accordance to Table 3 . The asterisk indicates nodes supported by a posterior probability of ≥0.95. The horizontal axis is expressed in calendar years. Branch colors indicate the origin of the sequences: blue, sequences from Equatoguinean patients (n = 39); green, sequences from Western Africa (n = 21); red, sequences from Central Africa (n = 17); black, other regions (n = 15). (TIF) [file pone.0064293.s001.tif]
