# Supplementary material for: Description of HIV-1 Group M Molecular Epidemiology and Drug Resistance Prevalence in Equatorial Guinea from Migrants in Spain
Source: PLoS One. 2013 May 22;8(5):e64293. doi: 10.1371/journal.pone.0064293 (PMC3661467; doi:10.1371/journal.pone.0064293)
Supplement: Table S1 — Sampling dates of the 92 CRF02_AG pol sequences included in the BEAST analysis. n, number of sequences. (DOCX) [file pone.0064293.s002.docx]

**Table S1.** Sampling dates of the 92 CRF02_AG *pol* sequences included in the BEAST analysis.

| **Sampling year** | **All sequences (n=92)** | **Equatoguinean sequences (n=39)** | **Other sequences (n=53)** |
| --- | --- | --- | --- |
| 1990 | 1 | 0 | 1 |
| 1991 | 1 | 0 | 1 |
| 1992 | 0 | 0 | 0 |
| 1993 | 2 | 0 | 2 |
| 1994 | 2 | 0 | 2 |
| 1995 | 1 | 0 | 1 |
| 1996 | 2 | 0 | 2 |
| 1997 | 8 | 7 | 1 |
| 1998 | 2 | 0 | 2 |
| 1999 | 3 | 1 | 2 |
| 2000 | 3 | 0 | 3 |
| 2001 | 4 | 1 | 3 |
| 2002 | 4 | 1 | 3 |
| 2003 | 3 | 0 | 3 |
| 2004 | 7 | 5 | 2 |
| 2005 | 9 | 6 | 3 |
| 2006 | 7 | 4 | 3 |
| 2007 | 11 | 3 | 8 |
| 2008 | 14 | 8 | 6 |
| 2009 | 3 | 1 | 2 |
| 2010 | 5 | 2 | 3 |

n, number of sequences.
