# Supplementary material for: Description of HIV-1 Group M Molecular Epidemiology and Drug Resistance Prevalence in Equatorial Guinea from Migrants in Spain
Source: PLoS One. 2013 May 22;8(5):e64293. doi: 10.1371/journal.pone.0064293 (PMC3661467; doi:10.1371/journal.pone.0064293)
Supplement: Accession S1 — GenBank accesion numbers of the 278 HIV-1 pol sequences included in this study. (DOCX) [file pone.0064293.s003.docx]

**ACCESSION NUMBERS**

Sequences sampled in Spain (n=195): AF125283(PR)/AF188336(RT), AF125287, AF125292(PR)/AF188342(RT), AF125293, AF125294(PR)/AF455646(RT), AF188349(PR)/AF188345(RT), AF247009, AF247012, AF247014, AF247017, AF247018 to AF247021, AF247023(PR)/AF455664(RT), AF247024, AF247025, AF247027, AF247028, AF247030, AF247033, AF354007(PR)/AF455638(RT), AF354012, AF354013, AF354025, AF354034(PR)/AF455634(RT), AF354038(PR)/AF455651(RT), AF354043(PR)/AF455647(RT), AF394957(PR)/AY050184(RT), AF455609(PR)/AF479601(RT), AF455620(PR)/AF479604(RT), AF455631, AF455665(PR)/AF455636(RT), AF455666(PR)/AF455637(RT), AY248292, AY248299, AY248309, AY248311, AY248313, AY248317, AY248325, AY248329, AY248331, AY248332, AY248431, AY642099, AY642101, AY642103, AY642106, AY642112, AY647440, AY647441-AY647443, AY647445-AY647447, AY647449, DQ009056, DQ009057, DQ157799, DQ157806, DQ157807, DQ157810, DQ157812, DQ157813, DQ157825, DQ157833, EF380366, EF380367, EF380381, EF421986, EU255306, EU255307, EU255309, EU255346, EU255349, EU255354, EU255355, EU255358, EU255360, EU255363, EU255373-EU255388, EU255465, EU255468, EU255485, EU255489, EU255507, EU255513, EU255522-EU255525, EU342758, EU342760, EU342761, EU342767, EU342772-EU342775, EU342778, EU342781, EU342784, EU342785, EU342795, EU342819, EU342825, EU362920, EU362923, EU362924, EU362926, EU545186, EU545187, EU545188, EU545190, EU545191, EU545193, EU545194, EU552227, FJ481668, FJ481693, FJ481701, GQ240994, GQ241042, GQ241050, GQ241052, GU264338, HM460493, HM460496, HQ426896, HQ426901, HQ426902, HQ426905, HQ426906, HQ426908, JF929046, JF929114, JF929133, JF929142, JQ351953, JQ351955, JQ351990 and JX428539-JX428575.

Sequences sampled in GQ (n=83): AF529922-AF529954, AY580058-AY580065, DQ157832 and FN557303-FN557343.
